# Induction of early Purkinje cell dendritic differentiation by thyroid hormone requires RORα

**DOI:** 10.1186/1749-8104-5-18

**Published:** 2010-07-27

**Authors:** Fatiha Boukhtouche, Bernard Brugg, Rosine Wehrlé, Brigitte Bois-Joyeux, Jean-Louis Danan, Isabelle Dusart, Jean Mariani

**Affiliations:** 1 UPMC Université Paris 6, UMR 7102 NPA, F-75005, Paris, France; 2 CNRS, UMR 7102 NPA, F-75005, Paris, France; 3 Biozentrum, Department of Cell Biology, University of Basel, CH-4056 Basel, Switzerland; 4 Faculté de Médecine Paris Descartes, site Necker, FRE CNRS 3210, F-75015, Paris, France; 5 Hôpital Charles Foix, UEF, F-94205, Ivry-sur-Seine, France

## Abstract

**Background:**

The active form (T_3_) of thyroid hormone (TH) controls critical aspects of cerebellar development, such as migration of postmitotic neurons and terminal dendritic differentiation of Purkinje cells. The effects of T_3 _on early dendritic differentiation are poorly understood.

**Results:**

In this study, we have analyzed the influence of T_3 _on the progression of the early steps of Purkinje cell dendritic differentiation in postnatal day 0 organotypic cerebellar cultures. These steps include, successively, regression of immature neuritic processes, a stellate cell stage, and the extension of several long and mature perisomatic protrusions before the growth of the ultimate dendritic tree. We also studied the involvement of RORα, a nuclear receptor controlling early Purkinje cell dendritic differentiation. We show that T_3 _treatment leads to an accelerated progression of the early steps of dendritic differentiation in culture, together with an increased expression of RORα (mRNA and protein) in both Purkinje cells and interneurons. Finally, we show that T_3 _failed to promote early dendritic differentiation in *staggerer *RORα-deficient Purkinje cells.

**Conclusions:**

Our results demonstrate that T_3 _action on the early Purkinje cell dendritic differentiation process is mediated by RORα.

## Background

The thyroid hormone (TH) L-3,3',5-triiodothyronine (T_3_) is essential for normal central nervous system development [1], regulating processes associated with brain differentiation, such as neuronal migration, axonal and dendritic growth, synaptogenesis, and myelination [2]. In particular, TH plays an important role in cerebellar neurogenesis [3-5], a mainly postnatal developmental process. As a consequence, perinatal hypothyroidism affects the morphogenesis of cerebellar neurons (in particular the dendritic arborization of the Purkinje cells (PCs), which display a striking reduction in the growth and branching of their dendritic arborization [6]) and delays synaptic formation between PCs and granule cells [3-5,7] (for review, see [8]). Recent studies have demonstrated that THs promote this growth of the PC mature dendritic tree through activation of the nuclear thyroid hormone receptor (TR) TRα1 [9,10].

Shortly after birth, cerebellar PCs display a bipolar shape reminiscent of their migratory morphology. These immature PCs then follow a process of dendritic regression, prior to extending dendrites from which the ultimate and characteristic mature dendritic tree will arise (for review, see [11]). We have recently demonstrated that the nuclear receptor Retinoic acid receptor-related orphan receptor alpha (RORα, NR1D1) controls the early dendritic differentiation steps, particularly the regressive phase of this process [12]. The loss-of-function *staggerer *(sg) mutation in the *Rora *gene leads to cerebellar developmental defects in the mouse, including dramatic PC and granule cell loss [13-16]. Interestingly, cross-talk between the TH pathway and RORα has been shown. In hypothyroid rats, daily thyroxine (T_4_) replacement accelerates the increase of RORα mRNA within the developing cerebellum, most obviously at P15 [17]. In the homozygous *staggerer *mutant mouse (*Rora^sg/sg^*), despite both normal TR expression [14] and normal serum TH levels [18], *staggerer *neurons seem to be unresponsive to TH treatment [19].

Despite the detailed description of cerebellar abnormalities due to hypothyroidism, most studies investigate the role of TH in the growth of the mature PC dendritic tree, which involves cross-talk and synaptogenesis with granule cells; but little is known about the effect of TH on early dendritic differentiation. In this study, we aimed at determining the role of TH in early PC dendritic differentiation, that is, during the phase of regression of the primary dendrite, and we studied the involvement of RORα in this process. Using organotypic cultures, we have thus studied the progression of PC dendritic differentiation in the presence or absence of T_3_, and we observed an acceleration of the process of dendritic differentiation when T_3 _was added onto postnatal day 0 (P0) cerebellar slices for both early events (regression of the primary dendrite observed after 3 days *in vitro *(DIV)) and later ones (growth of the mature dendritic tree). We further propose that the accelerated early dendritic differentiation is dependent on a T_3_-induced increase of RORα expression.

## Results

### Determination of the optimal T_3 _concentration to promote PC dendritic growth in organotypic culture

At birth, *in vivo*, most murine PCs are fusiform (bipolar shape, stage I; data not shown), as described for rats [20]. When cultured at P0 and kept in organotypic cultures, PCs present first an immature morphology (bipolar fusiform, stage I), then retract their primitive dendrites to become stellate or atrophic (stage II), elongate numerous long and mature dendritic perisomatic protrusions (stage III), and finally develop their ultimate dendritic trees (stage IV) [12].

In organotypic cultures, after 7 days in a serum-containing medium, PCs were mostly in stage II (stellate or atrophic stage), whereas almost no stage III PCs were observed. In order to explore the involvement of T_3 _in dendritic differentiation (that is, before the extension of the ultimate dendritic tree in stage IV), cultures from P0 animals were prepared and kept 7 DIV under serum-free conditions, with or without addition of T_3 _at different concentrations. Cultures were then fixed and immunolabeled with an anti-calbindin (anti-CaBP) antibody to visualize PCs.

**Figure 1:**
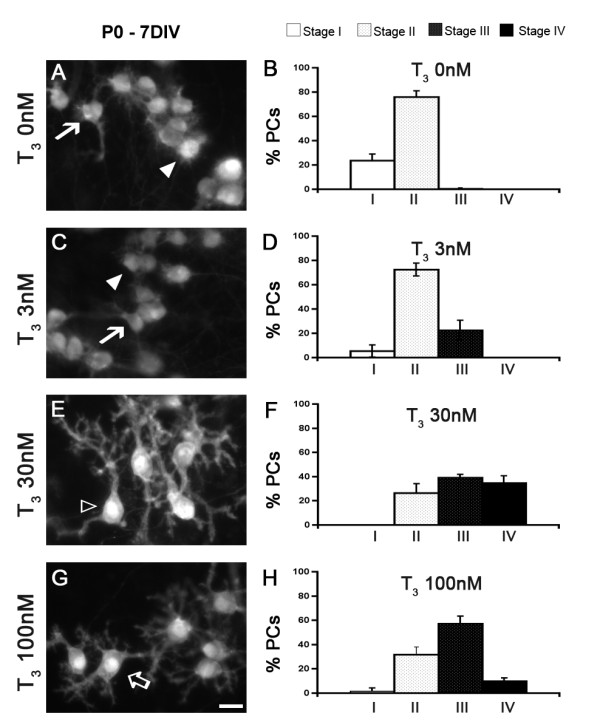
**Dose-dependent effect of T_3 _on PC dendritic differentiation in organotypic cultures**. **(A-H)**. Organotypic cultures of P0 cerebella were kept 7 DIV in the absence of T_3 _(A,B), or in the presence of 3 nM T_3 _(C,D), 30 nM T_3 _(E,F) or 100 nM T_3 _(G,H). (A,C,E,G) PCs were revealed by CaBP immunolabeling. (B,D,F,H) Quantitative distribution of PCs between stages I to IV. Fusiform PCs with a bipolar shape are defined as stage I (arrow in (A,C)), PCs with regressive atrophic dendrites all around the soma are defined as stage II (white arrowhead in (A,C)), PCs with one or more perisomatic protrusions are defined as stage III (empty arrow in (G)) and PCs with an identified dendritic tree are classified as stage IV (empty arrowhead in (E)). Scale bar = 20 μm. Error bars indicate mean ± standard deviation.

In P0 slices cultivated without T_3_, most PCs (76%) displayed 'stellate or atrophic' dendrites after 7 DIV (stage II; Figure 1A,B). Adding T_3 _at a concentration of 3 nM did not dramatically modify the repartition of PC classes (Figure 1C,D). In contrast, supply of 30 nM of T_3 _led to a significant acceleration of the dendritic differentiation since we observed that only 27% of PCs were in stage II, whereas 39% displayed long dendritic perisomatic protrusions and were thus classified as stage III PCs, and 34% displayed an identified mature dendritic tree (stage IV; Figure 1E,F). Increased concentration of T_3 _(100 nM) also led to an acceleration of the dendritic differentiation (compared to 0 nM and 3 nM of T_3_), although this treatment was not as efficient as 30 nM T_3 _since the proportion of stage IV PCs was lower (Figure 1G,H). Thus, these results show that, in organotypic culture, as already demonstrated for dissociated cell culture [10,11], the addition of T_3 _to the culture medium promotes PC dendritic development in a dose-dependent manner. In the following experiments, we have used the 30 nM concentration to assess the effects of T_3 _since this concentration is the most efficient in our culture conditions.

### T_3 _leads to an increased amount of RORα protein in PCs and interneurons

To determine whether the T_3_-induced acceleration of dendritic differentiation involves RORα, we first assessed RORα expression in cerebellar slices in response to T_3 _treatment. By combination of promoter usage and alternative splicing, the *Rora *gene encodes two isoforms in the mouse (RORα1 and RORα4), which differ only in their amino-terminal modulator region [21-23].

**Figure 2:**
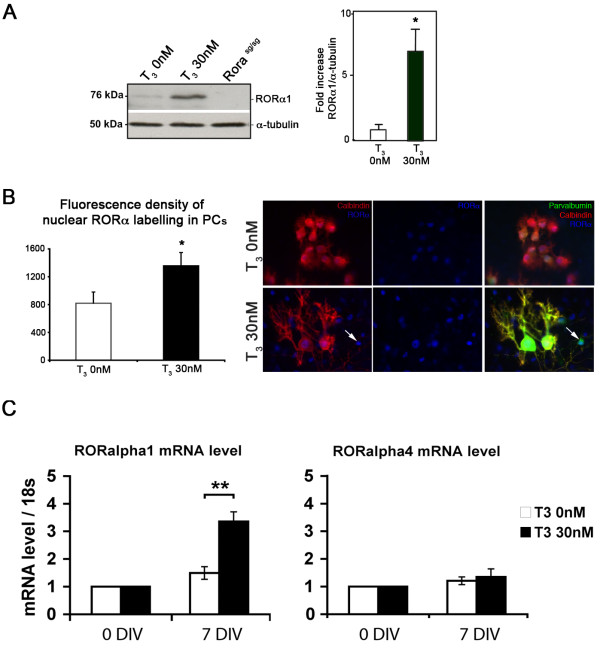
**T_3 _treatment increases the amount of RORα protein and RNA in organotypic cultures**. P0 cerebellar slices kept for 7 DIV were cultured in the absence or the presence of 30 nM T_3_. **(A) **Immunoblot analysis and quantification of RORα levels in extracts of untreated or T_3_-treated cerebellar slices (*P < 0.05). **(B) **Left panel: fluorescence density of RORα immunolabeling was measured within each PC nucleus with MetaMorph software. Average values from multiple cells ± SEM are shown (*P < 0.05). Right panel: organotypic cultures after 10 DIV without T_3 _(upper row) or with T_3 _(30 nM) treatment (lower row). RORα-expressing cells were revealed by RORα immunolabeling (blue), PCs were revealed by CaBP immunolabeling (red) and both PCs and interneurons were revealed by parvalbumin immunolabeling (green). Note the presence of RORα-expressing interneurons (arrow) in the T_3 _only treatment. **(C) **P0 organotypic cultures were cultured without T_3 _(white bars) or with 30 nM T_3 _(black bars) for 7 days. Levels of mRNA were determined by real time RT-PCR and standardized to 18 s rRNA The data are given relative to the mRNA level in untreated slices at the initial time of the culture (0 DIV). They were obtained from three independent cerebellar slices extracts (*P < 0.05; **P < 0.005). Error bars in (C) indicate mean ± standard deviation.

Western blots of P0 7 DIV cerebellar slices were performed with antibodies directed against the carboxyl terminus of RORα, which can detect both RORα1 and RORα4 isoforms. We detected an increase of 6.8-fold in the amount of RORα1 protein in slices treated with 30 nM of T_3 _(Figure 2A).

In the cerebellum, RORα is known to be expressed only in PCs and interneurons [24]. In order to determine in which cell type the upregulation of RORα expression occurs, we used immunofluorescence to detect and locate the RORα protein in organotypic cultures. Since only PCs in the cerebellum express CaBP, we used CaBP as a PC-specific marker, and we used parvalbumin as a marker of interneurons. Both mature interneurons and PCs express parvalbumin: interneurons were unambiguously identified as parvalbumin-positive and CaBP-negative cells.

To assess whether T_3 _led to increased expression RORα1 in PCs, we quantified the fluorescence density of RORα labeling within the nucleus of PCs. We observed a significant increase in the fluorescence density in T_3_-treated slices compared to control T_3_-untreated slices (Figure 2B). Interestingly, following T_3 _treatment, RORα labeling was observed in PCs and also in some CaBP-negative cells. Some CaBP-negative cells that express RORα also expressed parvalbumin, and were thereby identified as interneurons (Figure 2B). Therefore, T_3 _treatment led to increased expression of RORα in both PCs and parvalbumin-positive interneurons.

To determine whether the increase in RORα protein levels in T_3_-treated cerebellar slices is the consequence of increased expression of the *Rora *gene, and not stabilization of the protein, we analyzed by real time RT-PCR the mRNA level of the different *Rora *isoforms in the T_3_-treated slices compared to untreated slices after 7 DIV (Figure 2C). A 3.3-fold increase was observed for *Rora1 *after 7 DIV. The *Rora4 *mRNA level was similar to untreated slices after 7 DIV. These results show that T_3 _leads to increased expression of the *Rora1 *isoform after 7 DIV.

These results show that T_3 _induced increased RORα1 protein levels in PCs in parallel with their dendritic differentiation after 7 DIV.

### T_3 _accelerates the first steps of early PC dendritic differentiation and increases *Rora *gene expression at P0

**Figure 3:**
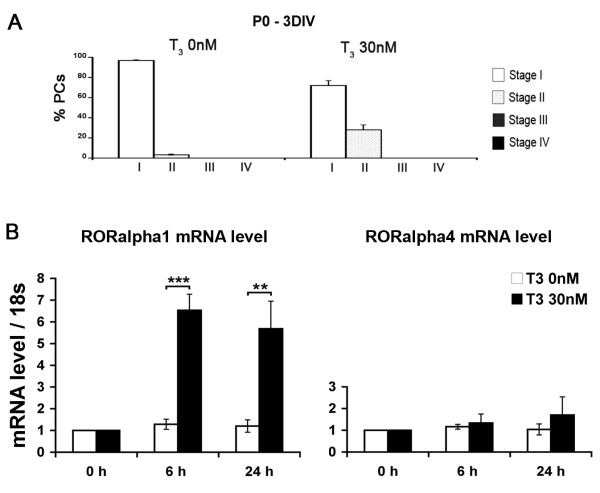
**T_3 _promotes the early dendritic differentiation of PCs and leads to increased mRNA levels of *Rora1 *and *Rora4 *at P0**. **(A) **Quantitative distribution of PCs between stages I and IV. Cultures of P0 cerebella were kept 3 DIV in the absence or the presence of 30 nM T_3_. PCs are classified following the same criteria as in Figure 1. **(B) **P0 organotypic cultures were cultured without T_3 _(white bars) or with 30 nM T_3 _(black bars). Levels of mRNA were determined by real time RT-PCR and standardized to 18 s rRNA after 0 h, 6 h or 24 h of T_3 _treatment. The data are given relative to the mRNA level in untreated slices at the initial time of the culture (0 h). They were obtained from three independent cerebellar slice extracts (**P < 0.005; *** *P *< 0.0005). Error bars indicate mean ± standard deviation.

As we previously demonstrated that RORα is involved in early dendritic differentiation [12], we examined whether T_3 _promotes this early change. We thus assessed the effect of T_3 _treatment on cerebellar slices after 3 DIV, a time when PCs cultured without T_3 _display mainly bipolar fusiform dendritic morphology (stage I; 97%) whereas very few are in a stellate or atrophic morphology (stage II; 3%; Figure 3A). In the presence of 30 nM of T_3 _after 3 DIV, all PCs were still in stage I or II, but we observed an increased number of PCs in stage II (28%; Figure 3A) compared to the control without T_3 _(3%; Figure 3A). From those experiments, we can conclude that T_3 _promotes the first dendritic differentiation steps of PCs from stage I to stage II in organotypic cultures.

To determine whether T3 increases *Rora *expression in early stages of PC development, we analyzed the mRNA levels of the *Rora *isoforms in response to T_3 _treatment during the first day of culture (Figure 3B). We observed a specific increase in the *Rora1 *mRNA level after 6 h and 24 h of T_3 _treatment (6.5- and 5.7-fold increase, respectively). The *Rora4 *mRNA level was slightly and transiently increased in slices after 24 h of T_3 _treatment (1.7-fold increase). These results show that T_3 _leads to increased expression of the *Rora1 *isoform in fusiform PCs at P0 and, to a lesser extent, of the *Rora4 *isoform. Interestingly, our results also revealed that mRNA levels of both *Rora1 *and *Rora4 *isoforms were stable in cultures made at P0 and kept for 6 h, 24 h and 7 DIV in culture without T_3 _(compare Figures 2C and 3B).

### T_3_-induced early dendritic differentiation involves RORα

The experiments described above show that T_3 _promotes dendritic differentiation (Figures 1 and 3) and leads to increased expression of RORα1 (Figures 2 and 3). We have recently shown that RORα is a crucial factor controlling the early steps of PC dendritic differentiation, and *staggerer *RORα-deficient PCs do not progress beyond early bipolar migratory morphology [12]. To determine whether RORα is actually involved in the T_3_-induced PC dendritic differentiation, we followed the progression of the dendritic differentiation of PCs from *staggerer *(*Rora^sg/sg^*) and corresponding control *Rora*^+/+ ^cerebellar slices treated or not with T_3_.

**Figure 4:**
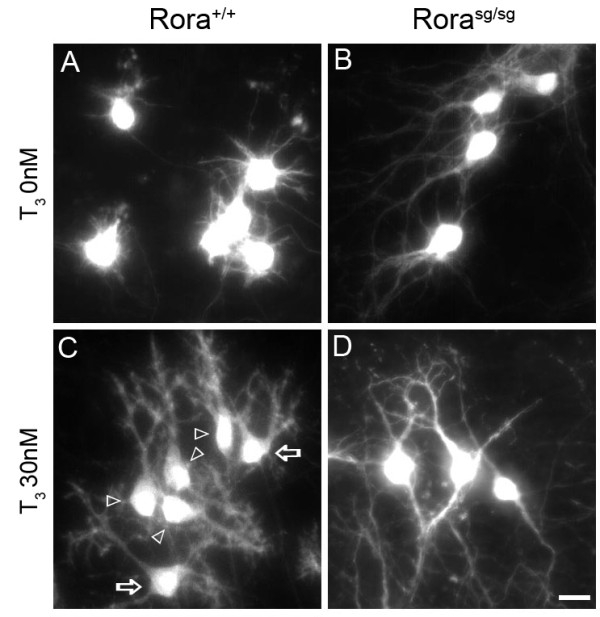
**T_3 _treatment fails to induce dendritic differentiation in early RORa-deficient *staggerer *PCs in organotypic cultures**. **(A-D) **PCs in organotypic cultures from wild-type (A,C) or RORα-deficient *Rora^sg/sg ^*(B,D) P0 mice were revealed by CaBP immunolabeling after 7 DIV without T_3 _(A,B) or with 30 nM T_3 _(C,D). PCs from wild-type mice responded to T_3 _treatment: without T_3_, PCs were mostly in stage II (PCs with regressive-atrophic dendrites all around the soma) whereas they were in stages II, III (empty arrows) and IV (empty arrowheads) after 30 nM T_3 _treatment (C). PCs from *Rora^sg/sg ^*mice were unresponsive to T3 treatment since they remain in stage I (fusiform PCs with long processes of bipolar shape) in the absence (B) or in the presence (D) of 30 nM T_3 _treatment. Note the bipolar or fusiform shape of the PCs with long processes but no dendritic arborization in (B,D). Scale bar = 20 μm.

As previously observed in serum-containing cultures [12], PCs from *Rora^sg/sg ^*cultured in serum-free medium display the embryonic bipolar shape (stage I) after 7 DIV (Figure 4B), whereas most PCs in control *Rora*^+/+ ^cultures display 'regressive-atrophic' dendrites (stage II; Figure 4A). In the presence of 30 nM T_3_, stage II, III and IV PCs were found (Figure 4C) with the same proportions as described in Figure 1. In contrast, PCs from *Rora^sg/sg ^*animals still display embryonic bipolar shape (Figure 4D) with long processes characteristic of stage I PCs [12], indicating that they were not responsive to T_3 _treatment and remained in the very early stage of dendritic differentiation.

The absence of a functional RORα protein thus prevents T_3_-induced accelerated dendritic differentiation of immature bipolar P0 PCs. This experiment shows that RORα is required in the T_3_-induced dendritic differentiation-promoting process.

### T_3 _up-regulates the activity of the *Rora *promoter

**Figure 5:**
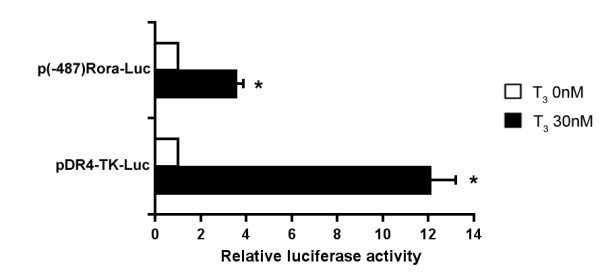
**T_3 _treatment upregulates *Rora *promoter activity**. HepG2 cells cultured in 12-well plates were co-transfected with 500 ng/well of the pTRα vector, allowing expression of the TR, and 500 ng/well of the p(-487)Rora-Luc reporter vector, which allows expression of the luciferase gene under the control of the human genomic sequences between nucleotides -487 and -45 from the *Rora1 *translation initiation site, or with 500 ng/well of the promoter-less pGL3-Luc vector. HepG2 cells co-transfected with 500 ng/well of the pTRα vector and of the pDR4-TK-Luc reporter vector were used as a control of the transcriptional effect of T_3 _on a TH response element. The luciferase activity of p(-487)Rora-Luc and pDR4-Luc in the absence or the presence of T_3 _(30 nM) is expressed relative to that of the promoter-less pGL3-Luc vector (*P < 0.05). Error bars indicate mean ± standard deviation.

To gain further insight into the mechanism by which T_3 _up-regulates *Rora *gene expression, we tested the effect of T_3 _on the transcriptional activity of the p(-487)Rora-Luc construct in HepG2 cells, which shows 82.9% sequence homology with the murine sequence and has been previously used as a model to analyze the transcriptional regulation of the *Rora *gene [25] (Additional file 1). T_3 _treatment resulted in a 3.6-fold increase in the activity of the p(-487)Rora-Luc construct compared to its basal activity in the absence of T_3 _(Figure 5). Plasmid pDR4-TK-Luc, which contains a thyroid receptor response element (DR4), was used as a control for the effect of T_3 _on transcriptional activity. As expected, the luciferase activity of pDR4-TK-Luc was strongly increased (12.1-fold) by T_3 _treatment, indicating that T_3 _is active under our experimental conditions.

Taken together, the results of these experiments indicate that the effect of T_3 _on *Rora *expression is, at least in part, transcriptional and that the -487 to -45 *Rora *promoter region is involved in this regulation.

## Discussion

Our results show for the first time that T_3 _promotes the early steps of PC dendritic differentiation, during the phase of neurite regression that precedes the formation of the ultimate dendritic tree. Addition of T_3 _to the serum-free medium of P0 cerebellar slices resulted in an acceleration of the early steps of dendritic differentiation. This accelerated progression of dendritic differentiation was accompanied by increased expression of the gene encoding the nuclear receptor RORα, observed at both mRNA and protein levels. We further show that the RORα protein is required for the T_3_-induced early dendritic differentiation, as T_3 _treatment did not promote dendritic differentiation in *Rora^sg/sg ^*PCs. This result is in accordance with previous studies that suggest an unresponsiveness of *Rora^sg/sg ^*mutants to TH [19].

### T_3 _promotes early PC dendritic differentiation

The role of TH in mammalian brain is well documented, particularly during cerebellar development (for review, see [1,26]). Congenital hypothyroidism in humans leads to a syndrome termed cretinism [27], the apparent symptoms of which include ataxia and poor motor skills, indicating cerebellar defects. In PCs, TH is known to strongly promote differentiation of the elaborate dendritic tree and synapse formation. In contrast, little is known about its role in the events preceding the development of the ultimate dendritic tree, in particular the steps of neuritic regression and early extension of perisomatic protrusions, occurring *in vivo *in the rodent between P0 and P7.

To better understand the effect of TH action in the developing brain, the temporal patterns of initiation and cessation of hormone action need to be determined. Most *in vitro *or *in vivo *experiments explore the effects of hypo- or hyperthyroidism in the cerebellum from P15, or its equivalent age in culture. At this age, the characteristic shape of the dendritic arborization is already achieved, and extrinsic factors such as electrical activity [28] from granule cells, trophic factors [29-32] and TH modulate the growth of the dendritic arborization [4]. Studies have shown a role for TH in the persistence of the external granular layer and the migration of granule cells into the internal granular layer [4,5,33], in the proliferation and differentiation of interneurons [34], as well as a direct role of TH on PCs through TRα1 receptor activation [10]. However, the effects of TH on PC dendritic differentiation during early steps that do not require cell-cell interaction have not been shown.

Using P0 slices after 3 DIV, we could specifically assess the role of TH in early development, and our results show that T_3 _also plays a key role in the early dendritic differentiation of PCs in organotypic cultures, that is, before the formation of the elaborated dendritic tree. These data extend the well-known role of T_3 _in the later stages of PC dendritic differentiation [6,8-10] and identify a third molecule besides RORα and SCLIP [12,35] involved in the first steps of PC dendritic differentiation. Interestingly, T3 is the first extrinsic factor described to play a role in these processes.

We also show that T_3 _promotes the expression of both RORα and parvalbumin in interneurons, which corroborates recent results from Manzano *et al. *[34], who have shown that TH acts on the proliferation and differentiation of interneuron precursors in the cerebellum [34]. Further studies will be required to determine whether the TH action on interneurons is mediated by RORα.

### Cross-talk between RORα and the TH pathway

Interestingly, T_3 _addition led to increased expression of RORα in the cerebellar PCs and interneurons. This result is in accordance with previous studies that showed decreased expression of RORα in the cerebellum of hypothyroid rats [36], whereas T_4 _replacement led to increased expression [17].

Our results indicate that RORα1 expression is required for the T_3_-induced effect on early dendritic differentiation. Further, we show that the activity of the *Rora *promoter was enhanced by T_3 _treatment in culture, suggesting that TH acts on the process of early dendritic differentiation through increased expression of the *Rora *gene. TH binds to the nuclear TH receptor (TR), a ligand-regulated transcription factor, which then binds to a target DNA sequence known as a TH response element (TRE) within the promoter region of target genes. Further studies are needed to determine whether *RORα *is a target gene of TR, and whether the transcriptional effect observed in our study is under direct control. An additional level of interaction between RORα and TR has been demonstrated by Koibuchi and collaborators [37,38], who showed that RORα1 increases TR-induced transactivation on several TREs. This could account for the RORα requirement in the T_3_-mediated promotion of PC dendritic differentiation observed in this study. TR binds as a monomer, homodimer, or heterodimer (particularly with retinoid × receptors) to the TRE, which is composed of two half-site core motifs (AGGTCA) with specific nucleotide spacing and orientation. RORα binds as a monomer to a consensus motif composed of a 6-bp AT-rich sequence 5' to a half-site core motif, AGGTCA (ROR-response element, RORE), to activate transcription [23]. Both TR and RORα are thus transcription factors that share the common core motif within their response elements. RORα1 is able to bind as a monomer to one of two core motifs (AGGTCA) of a TRE that is preceded by an AT-rich sequence [23,37]. This suggests that a subset of natural TREs containing appropriate AT-rich sequences could serve as dual-response elements for TR and RORα. Because of the high homology between the human and murine RORα1 coding and promoter sequences, it is possible that RORα mediates some TH actions in human. Beside its roles in the developing cerebellum, RORα has also been shown to play critical roles in many different tissues and systems, including immunity, cancer, cellular metabolism, circadian rhythm, development and ageing (for review, see [39]). Understanding the roles of RORα could therefore provide further information about the pleiotropic effects of late prenatal or early postnatal hypo- and hyperthyroidism in humans.

### Intrinsic effect of RORα, and potential coordination with TH in the PC dendritic differentiation process

RORα has been shown to be crucial for the progression of early differentiation of PCs in a cell-autonomous manner [12]. In cerebellar slices, T_3 _is likely to act on RORα expression within PCs. Our results extend previous studies of Heuer and Mason [10], which clearly demonstrated that PCs are a direct target of TH action through activation of TRα1: TH promotes the late stages of the elaboration of PC dendritic arborization, which is also dependent upon granule cell differentiation and synaptogenesis. Interestingly, RORα has been shown to control the expression of *Sonic hedgehog *(*Shh*) in PCs, which in turn promotes the proliferation of granule cells precursors in the external granular layer [40]. Thus, a coordinate mechanism involving RORα and TH in cerebellar development can be proposed in which both T_3 _and RORα act on PC dendritic differentiation directly as well as indirectly via the promoting effect on granule cell development. However, the later and direct effects of T_3 _on early PC differentiation are unlikely to be mediated by RORα since we have shown that RORα does not influence this later step of differentiation [12].

In its homozygous state, the murine *staggerer *mutation of the *Rora *gene leads to cerebellar atrophy due to the degeneration of most PCs [13,15,41-43]. Several histological studies of the *Rora^sg/sg ^*cerebellum show that the remaining PCs are immature and display atrophic dendrites, devoid of spines [44-46]. These abnormalities of dendritic differentiation observed in homozygous *staggerer *mice are similar to, but worse than, those observed in hypothyroid rats. This implies that RORα acts on additional processes in cerebellar development, apart from those induced by THs. This hypothesis is strengthened by the recently demonstrated neuroprotective role of RORα at least partly through its control of oxidative stress mechanisms [16,47].

In conclusion, our results show that RORα plays a critical role in the early T_3_-induced dendritic differentiation of PCs.

## Materials and methods

### Animals

Animal housing and all procedures were carried out in accordance with the guidelines of the French Ministry of Agriculture and the European Community. Swiss mice were obtained from Janvier (Le Genest-St-Isle, France). The *staggerer Rora^sg/sg ^*mutant mice were maintained on a C57BL/6J genetic background in our colony. *Rora^sg/sg ^*and their *Rora*^+/+ ^littermates were obtained by intercrossing fertile heterozygous *Rora*^+/*sg *^animals, and were genotyped by PCR, as previously described [12].

### Organotypic slice cultures

Swiss mice at P0 were used. Organotypic cultures of cerebellum were prepared as described previously [48]. Briefly, after decapitation, brains were dissected out into cold Gey's balanced salt solution (Sigma, Lyon, France) supplemented with 5 mg/ml glucose, and the meninges were removed. Parasagittal cerebellar slices (350 μm thick) were cut on a McIlwain tissue chopper (Stoetling Europe, Dublin, Ireland) and transferred onto 30 mm Millipore membrane culture inserts with a 0.4 μm pore size (Millicell CM, Millipore, Molsheim, France). Slices were maintained in culture in six-well plates containing 1 ml per well of medium containing basal medium with Earle's salts (BME), supplemented with Sigma I-1884 supplement (1:100 dilution, resulting in final concentrations of 5 μg/ml insulin, 5 μg/ml transferrin, and 5 ng/ml sodium selenite), 0.5 μg/ml BSA (Sigma), 4 mM L-glutamine (Invitrogen, GIBCO, Cergy Pontoise, France), 5 mg/ml glucose, with or without T_3 _at 37°C in a humidified atmosphere with 5% CO_2_. The medium was replaced every 2 days (after 2, 4 and 6 days in culture).

Mice obtained from *Rora*^+/*sg *^intercrosses were also used in this study. In these litters, *Rora*^+/+^, *Rora*^+/*sg *^and *Rora^sg/sg ^*mice could be generated. For each animal, slices of each cerebellum were divided between two Millicells: half of the cerebellar slices served as controls and no T_3 _was added and the other half were treated with T_3 _(30 nM) in order to compare control (0 nM T_3_) versus T_3_-treated slices (30 nM T_3_) from the same animals. The genotype was determined *a posteriori *by PCR on tail biopsy, in blind studies.

### Antibodies and staining procedures

Immunostaining of CaBP, parvalbumin or RORα was performed as described previously [12]. Briefly, cerebellar slices were fixed in 4% paraformaldehyde, and then incubated for 1 h in phosphate-buffered saline containing 0.25% Triton X-100, 0.2% gelatin, 0.1% sodium azide (PBSGTA) and 0.1 M lysine. Rabbit polyclonal or mouse anti-CaBP antibody (1:5,000 dilution; Swant, Switzerland) to visualize PCs, or rabbit polyclonal anti-parvalbumin (1:5,000 dilution; Swant) to visualize both PCs and interneurons, and goat polyclonal anti-RORα1 (sc-6062; 1:4,000 dilution; Santa-Cruz, Tebu-Bio SA, Le Perray en Yvelines, France) in PBSGTA were applied overnight. At this dilution, the intensity of RORα labeling was correlated to the RORα expression level [12]. Specific labeling was detected with Cy3-conjugated donkey anti-rabbit antibody (1:500 dilution; Jackson Immunoresearch, Immunotech, Marseille, France) and FITC-conjugated donkey anti-goat antibody (1:2,000 dilution; Jackson Immunoresearch). The slices were analyzed with an inverted microscope (Nikon Eclipse TE 300). Immunofluorescence images were captured at 400× magnification using a Qimaging Retiga 1300 camera, and analyzed using Image-Pro Plus 4.1 software (Media Cybernetics, Bethesda, MD, USA). For RORα fluorescence intensity measurements, fluorescence density was measured in the nucleus of PCs (visualized by CaBP immunolabeling) using MetaMorph software.

### Classification of PC dendritic differentiation stages

Classification of PCs was assessed after CaBP immunostaining, as previously described [12]. Briefly, fusiform PCs with a bipolar shape, reminiscent of embryonic migratory PCs, are defined as stage I and correspond to both 'simple' and 'complex' fusiform stages, observed from embryonic day 16 to P4 *in vivo *[20]. Stage II comprises PCs with short processes all around the soma. This 'stellate' stage includes both 'regressive-atrophic dendrites' and 'stellate cell' stages described previously, from P2 to P6 *in vivo*. PCs with more than one long and mature dendritic protrusion are defined as stage III. They correspond to PCs around P5 to P10 *in vivo*. Finally, PCs with one well identified dendritic tree (defined as primary dendrites giving rise to additional side branches) are classified as stage IV. Images were taken from all slices, corresponding to at least 200 PCs in each experiment. Quantification was performed on three independent experiments.

### Western-blot analysis

Cultured slices were lysed in solubilization buffer (500 mM NaCl, 1 mM MgCl_2_, 2 mM EGTA, 50 mM Bicine, pH 9.0, 50 mM NaF, 5 μM ZnCl_2_, 100 μM Na_3_VO_4_, 1 mM dithiothreitol, 5 nM okadaic acid, 2.5 μg/ml aprotinin, 3.6 μM pepstatin, 0.5 μM phenylmethylsulfonyl fluoride, 0.5 mM benzamidine, 5.3 μM leupeptin) and dounced at 4°C. Insoluble materials were removed by centrifugation (13,000 g for 20 minutes at 4°C), supernatants were isolated and the samples were stored at -80°C. Proteins were dosed with the DC protein assay kit (Bio-Rad, Hercules, CA, USA). As previously described [49], cell-extracts containing equivalent amounts of protein were boiled for 5 minutes in sample loading buffer. After a 10% SDS-PAGE, proteins were transferred to a polyvinylidene difluoride membrane (ICN Biochemicals, Costa Mesa, CA USA). Non-specific sites were blocked with 5% skimmed dried milk for 2 h. Blots were then incubated overnight at 4°C with primary antibodies against RORα (1:2,000; Santa Cruz) and α-tubulin (1:10,000; Sigma) in 5% skimmed dried milk. They were then incubated with horseradish peroxidase-conjugated secondary antibodies in 5% skimmed dried milk for 1 h. The revelation was processed with enhanced chemoluminescence substrate (Amersham, Saclay. France). Quantification was performed using Densylab software (Microvision Instruments, Evry, France).

### Real-time RT-PCR

Total RNA from cerebellar slices from three animals was prepared according to the manufacturer's instructions using the RNeasy kit (Qiagen, Courtaboeuf, France) and cDNAs were synthesized from 1 μg of RNA (Promega, Charbonnieres-les-Bains, France) and avian myeloblastosis virus (AMV) reverse transcriptase, as per the manufacturer's instructions.

RT-PCR was performed using the ABsolute™ QPCR SYBR^® ^Green Mixes Kit (ABgene, Courtabeoeuf, France), as per the manufacturer's instructions. Reactions were performed in 25 μl of total volume containing ABsolute™ QPCR SYBR^® ^Green Mix with 8 ng of the first-strand cDNA and 300 nM of primers. The following primers were used: *Rora1 *sense, 5'-AGGCAGAGCTATGCGAGC-3', and antisense, 5'-TCAAACAGTTCTTCTGACGAGG-3'; *Rora4 *sense, 5'-GTCACATGGAGCCTCTTATGG-3', and antisense, 5'-TCAAACAGTTCTTCTGACGAGG-3'; 18 s sense, 5'-GGGAGCCTGAGAAACGGC-3', and antisense, 5' GGGTCGGAGTGGGTAATTT-3'. Amplification was performed on an iCycler (Bio-Rad) according to the manufacturer's instructions and cycle parameters were: 50°C (2 minutes) and 95°C (10 minutes), followed by 40 cycles of 95°C (15 s), 60°C (30 s) and 72°C (30 s). For expression quantification, a comparative *C*_T _method was used [50,51]. The Δ*C*_T _value was obtained by subtracting the *C*_T _value of the 18 S (reference) from the *C*_T _value of the gene of interest, where in each case the mean value of three reactions was used. For each gene, the fold change was calculated according to the formula , where ΔΔ*C*_T _was the difference between the Δ*C*_T _of T3-treated cultures and the Δ*C*_T _of untreated cultures as a calibrator value. To distinguish specific amplicons from non-specific amplifications, a dissociation curve was generated for each transcript. Quantification was performed on three independent experiments.

### Vectors, transient transfection and luciferase assay

The plasmid p(-487)Rora-Luc contains the luciferase reporter gene placed under the control of the promoter region of the human *Rora *gene, from -487 to -45 relative to the *Rora *translation initiation site [25]. The vector pTRα, containing mouse *TRα1 *cDNA, cloned in plasmid pSG5 and plasmid pDR4-TK-Luc, which contains a TRE in front of the promoter of the thymidine kinase gene of the herpes simplex virus controlling expression of the luciferase gene where kind gifts of Dr F Flamand (Ecole Normale Superieure, Lyon, France).

The promoter-less pGL3-basic luciferase reporter vector (pGL3-Luc) was from Promega. Transient transfection experiments were done with HepG2 human hepatoma cells using the calcium phosphate method. Twenty-four hours after the transfection, 30 nM of T_3 _were added to the medium and the luciferase activity was assayed 24 h later, as described [25]. Activities corresponding to cells cultured with 30 nM of T_3 _were expressed relative to those of control cells cultured without T_3_.

### Statistical analysis

Independent experiments were performed with 10 to 12 cerebellar slices per sample and repeated three times using matched controls. For PC stage quantification, at least 200 PCs were analyzed in each sample. For the RORα RNA level quantification by real-time PCR, all slices of three animals were used in each experiment. Results are expressed in Figures as mean ± standard deviation. The statistical significance of differences between control and T_3_-treated slices was assessed by a Student's *t*-test using Statview software (SAS Institute Inc., Berkeley, CA, USA).

## Abbreviations

bp: base pair; CaBP: calbindin; DIV: days *in vitro*; P: postnatal day; PC: Purkinje cell; RORα: Retinoic acid receptor-related orphan receptor alpha; sg: staggerer; T_3_: L-3,3',5-triiodothyronine; TH: thyroid hormone; TR: thyroid hormone receptor; TRE: TH response element.

## Competing interests

The authors declare that they have no competing interests.

## Authors' contributions

FB conceived of the study, designed and conducted experiments, and wrote the manuscript. RW and BBJ contributed to experiments; BB contributed to experiments and helped edit the manuscript. JLD, ID and JM supervised the study, and participated in its design and coordination, and helped edit the manuscript.

## Supplementary Material

Additional file 1 **Sequence comparison of the human and mouse *RORa1 *promoter region**. Human and mouse sequences of the immediately upstream region of the translation initiation codon (+1) of the *Rora *gene and including the -487 to -45 *Rora *promoter region (boxed area) were aligned using ClustalW software. The sequence downstream of the initiation codon corresponds to the beginning of exon 1. The nucleotide sharing identity across both species are indicated by asterisks and gaps are indicated with hyphens. The -487 to -45 human sequence shows 82.9% identity across species. Both human and murine sequences were obtained from the GenBank database: *Homo sapiens *chromosome 15 genomic contig [NT_010194.17], 32312505 to 32311809 bp; *Mus musculus *chromosome 9 genomic contig, strain C57BL/6J [NT_039474.7|Mm9_39514_37], 14921696 to 14922363 bp). Click here for file
