# Supplementary figures and images for: Induction of early Purkinje cell dendritic differentiation by thyroid hormone requires RORα
Source: Neural Dev. 2010 Jul 27;5:18. doi: 10.1186/1749-8104-5-18 (PMC2918593; doi:10.1186/1749-8104-5-18)

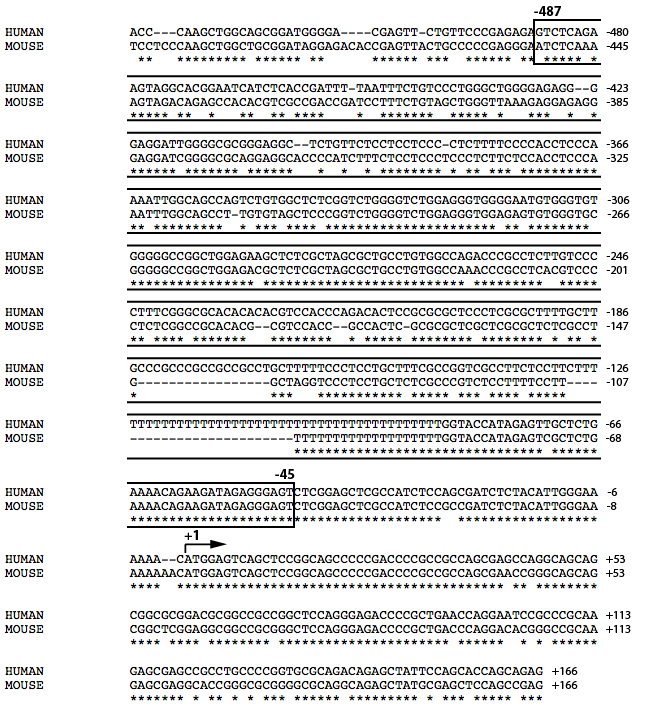

Supplement: Additional file 1 — Sequence comparison of the human and mouse RORa1 promoter region. Human and mouse sequences of the immediately upstream region of the translation initiation codon (+1) of the Rora gene and including the -487 to -45 Rora promoter region (boxed area) were aligned using ClustalW software. The sequence downstream of the initiation codon corresponds to the beginning of exon 1. The nucleotide sharing identity across both species are indicated by asterisks and gaps are indicated with hyphens. The -487 to -45 human sequence shows 82.9% identity across species. Both human and murine sequences were obtained from the GenBank database: Homo sapiens chromosome 15 genomic contig [NT_010194.17], 32312505 to 32311809 bp; Mus musculus chromosome 9 genomic contig, strain C57BL/6J [NT_039474.7|Mm9_39514_37], 14921696 to 14922363 bp). [file 1749-8104-5-18-S1.TIFF]
